# Supplementary material for: Modulating the strong metal-support interaction of single-atom catalysts via vicinal structure decoration
Source: Nat Commun. 2022 Jul 22;13:4244. doi: 10.1038/s41467-022-31966-1 (PMC9307766; doi:10.1038/s41467-022-31966-1)
Supplement: Supplementary file 1 — Supplementary information [file 41467_2022_31966_MOESM1_ESM.pdf]

Modulating the strong metal-support interaction of single-atom catalysts via vicinal structure decoration

Jingyi Yang,[1,ǂ] Yike Huang,[1,2,ǂ] Haifeng Qi,[1] Chaobin Zeng,[3] Qike Jiang,[4] Yitao Cui,[5] Yang Su,[1] Xiaorui Du,[1] Xiaoli Pan,[1] Xiaoyan Liu,[1] Weizhen Li,[1,*] Botao Qiao,[1,4,*] Aiqin Wang,[1] Tao Zhang[1,6]

[1] CAS Key Laboratory of Science and Technology on Applied Catalysis, Dalian Institute of Chemical Physics, Chinese Academy of Sciences, Dalian 116023 (China)

[2] University of Chinese Academy of Sciences, Beijing 100049 (China)

[3] Hitachi High Technologies (Shanghai) Co., Ltd, Shanghai 201203 (China)

[4] Dalian National Laboratory for Clean Energy, Chinese Academy of Sciences, Dalian 116023 (China)

[5] Synchrotron Radiation Laboratory, Laser and Synchrotron Research Center (LASOR), The Institute for Solid State Physics, The University of Tokyo, 1-490-2 Kouto, Shingu-cho Tatsuno, Hyogo 679-5165 (Japan)

[6] State Key Laboratory of Catalysis, Dalian Institute of Chemical Physics, Chinese Academy of Sciences, Dalian 116023 (China)

[ǂ] These authors contributed equally: Jingyi Yang, Yike Huang.

* Corresponding authors: W. Li (weizhenli@dicp.ac.cn); B. Qiao (bqiao@dicp.ac.cn)

Supplementary Figures

Figure S1

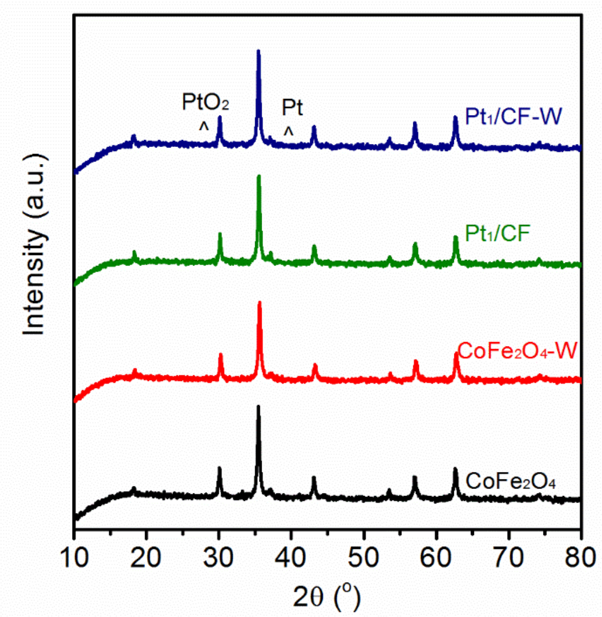

Figure S1. XRD patterns of the $CoFe_2O_4$ support and Pt_1/CF catalysts before and after water treatment.

Only diffraction peaks of $CoFe_2O_4$ were observed on the $CoFe_2O_4$, $CoFe_2O_4$-W, Pt_1/CF and Pt_1/CF-W samples, suggesting that the loading of Pt and water treatment do not induce phase transformation of the $CoFe_2O_4$ spinels. No Pt or PtO_2 diffraction peaks were found on all the spectrum which illustrates the finely dispersed Pt species on Pt_1/CF and Pt_1/CF-W.

Figure S2

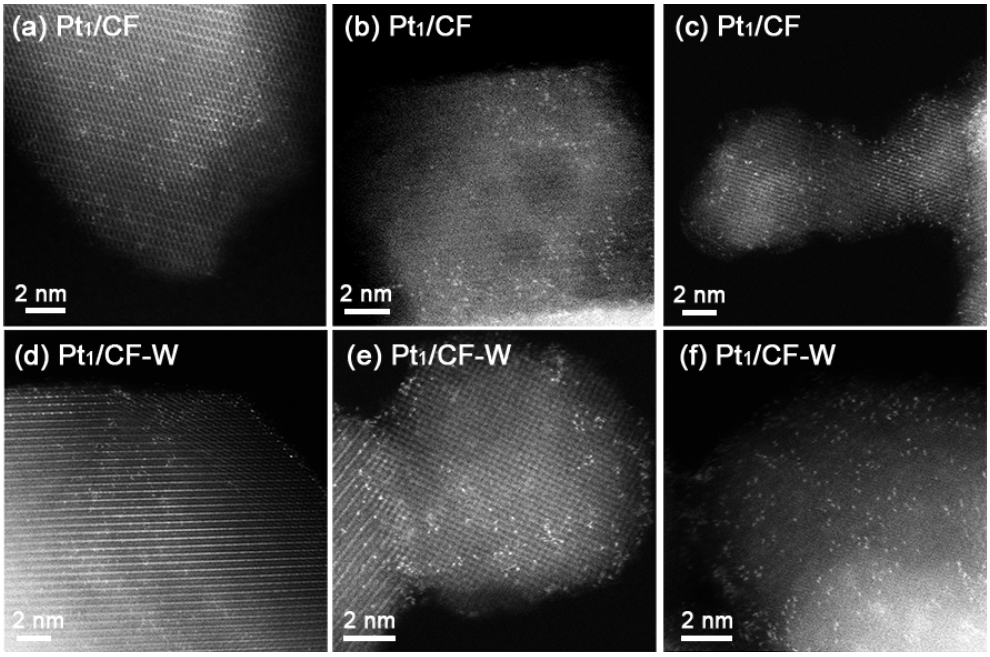

Figure S2. HAADF-STEM images of 1 wt% Pt_1/CF catalysts before and after water treatment: (a-c) Pt_1/CF and (d-f) Pt_1/CF-W.

Figure S3

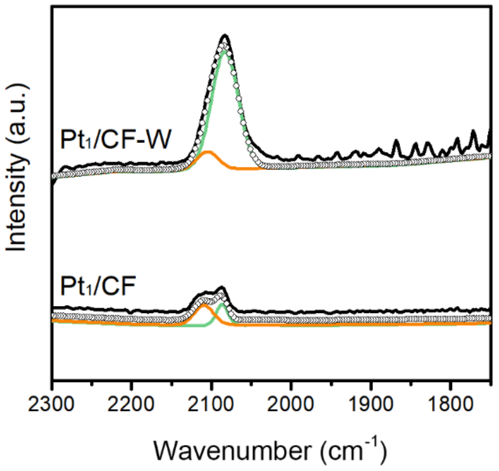

Figure S3. CO-DRFIT spectra of Pt_1/CF and Pt_1/CF-W.

Figure S4

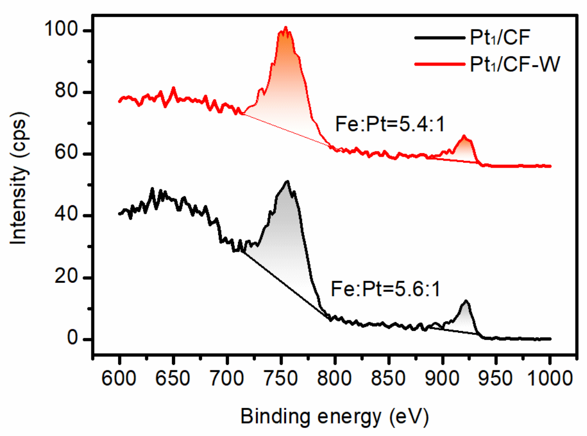

Figure S4. Ion scattering spectroscopy of Pt_1/CF and Pt_1/CF-W.

Figure S5

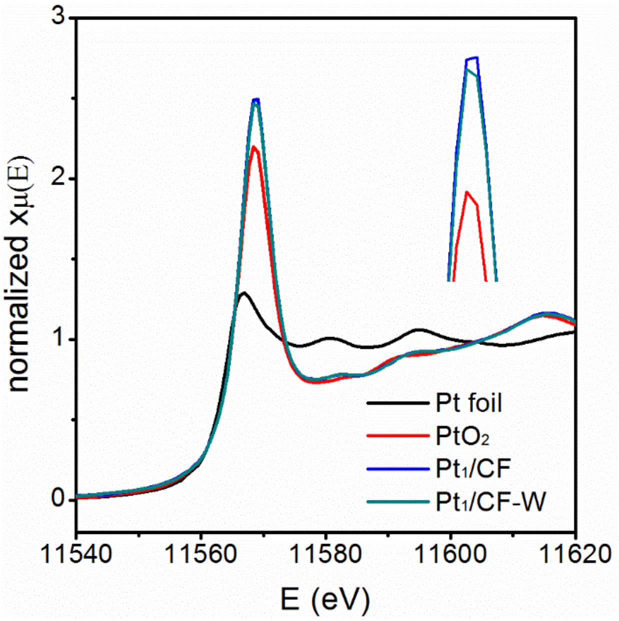

Figure S5. The normalized XANES spectra at the Pt L_3-edge.

Figure S6

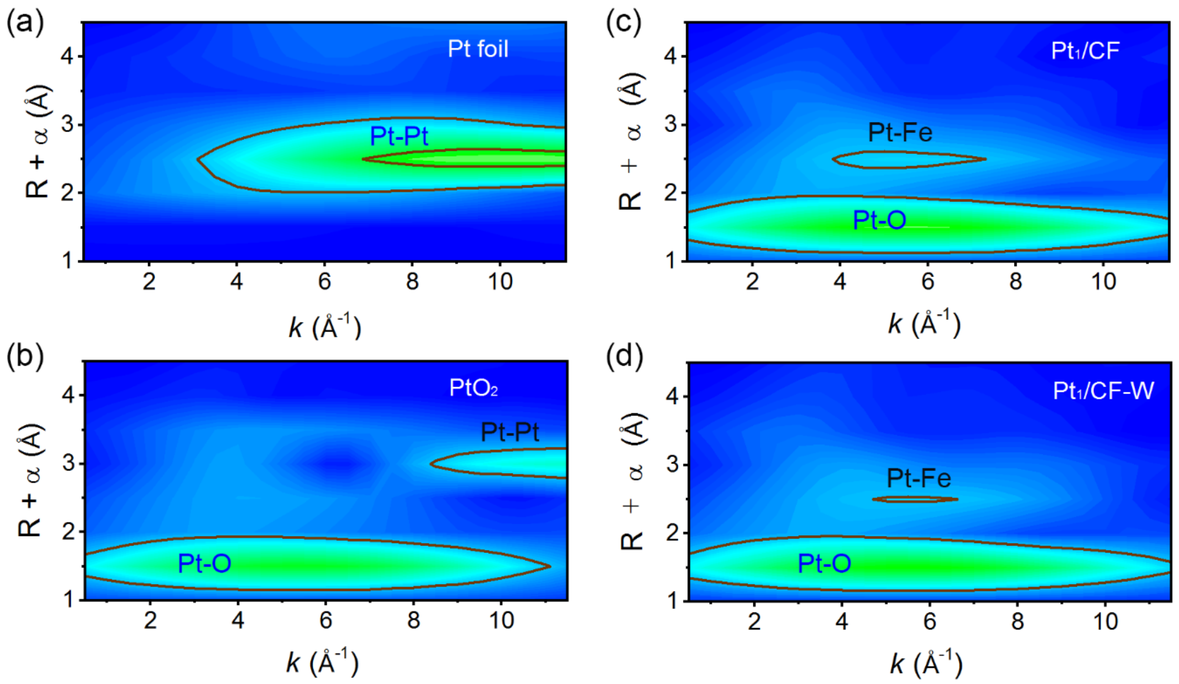

Figure S6. Wavelet transform of EXAFS: (a) Pt foil, (b) PtO_2, (c) Pt_1/CF and (d) Pt_1/CF-W.

Figure S7

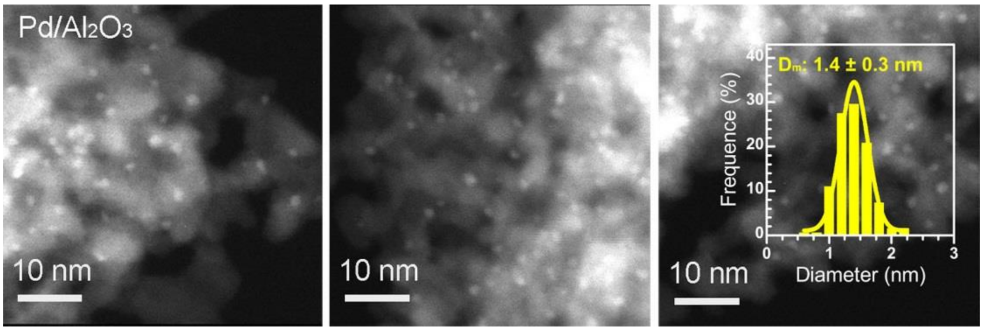

Figure S7. HAADF-STEM images of 1 wt% Pd/Al_2O_3. The inset is the size distribution of Pd nanoparticles.

The average size of Pd nanoparticles is 1.4 nm.

Figure S8

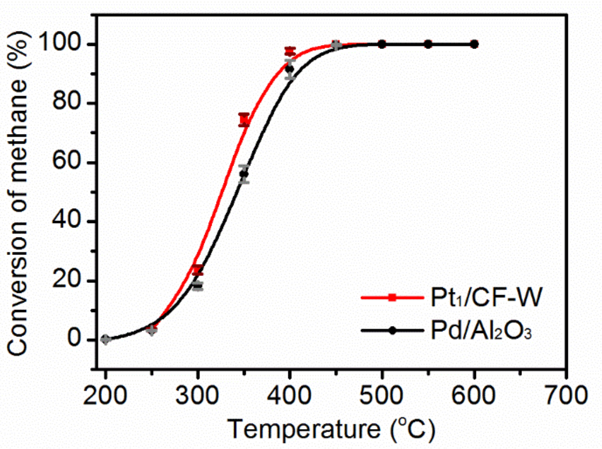

Figure S8. Light-off curves of CH_4 combustion over Pt_1/CF-W and Pd/Al_2O_3 catalysts. The reaction conditions: 0.5%CH_4, 20%O_2 balanced with N_2. GHSV = 20,000 ml/(g_{cat}·h). The error bars represent the standard deviation based on four repeated light-off experiment.

Figrue S9

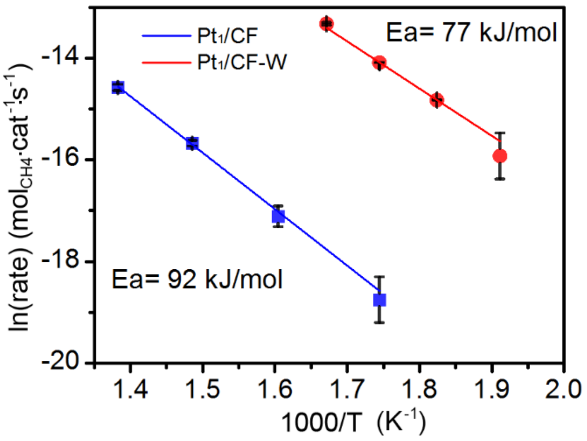

Figure S9. Arrhenius-type plots of methane combustion kinetics. All experiments were conducted with 0.5 vol% CH_4, 20 vol% O_2, and 79.5 vol% N_2. The GHSV was 20,000 for Pt_1/CF; the GHSV was 80,000 for Pt_1/CF-W. The error bars represent the standard deviation of three experiment.

Figrue S10

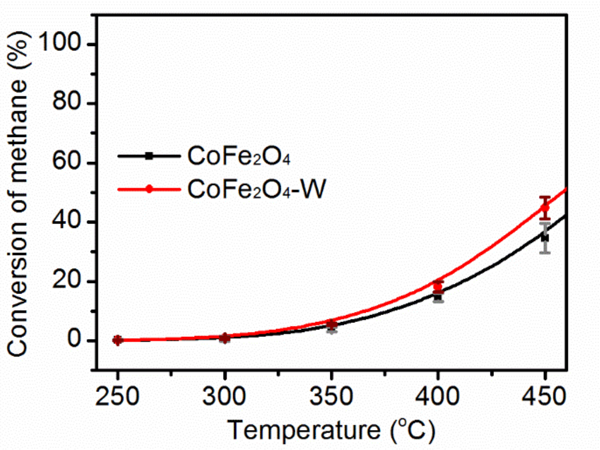

Figure S10. Light-off curves of CH_4 combustion over $CoFe_2O_4$ and $CoFe_2O_4$-W. The reaction conditions: 0.5%CH_4, 20%O_2 balanced with N_2, GHSV = 20,000 ml/(g_{cat}·h). The error bars represent the standard deviation based on four repeated light-off experiment.

The activity of $CoFe_2O_4$ and $CoFe_2O_4$-W are very limited below 450 °C, while the complete CH_4 conversion temperature is around 400 °C on the Pt_1/CF-W catalysts.

Figure S11

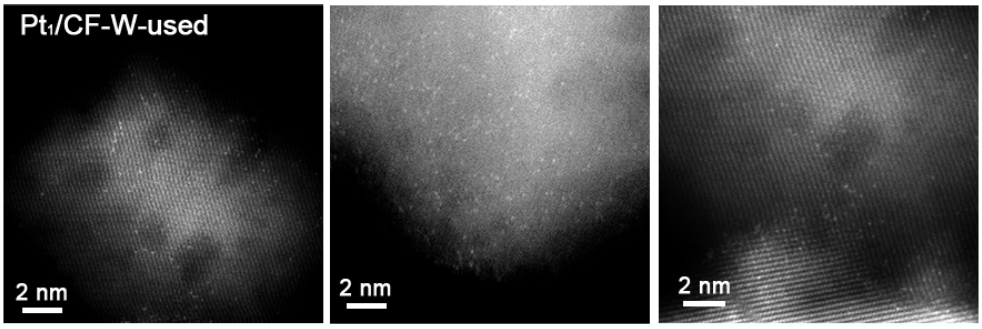

Figure S11. HAADF-STEM images of the Pt_1/CF-W after reaction.

Figure S12

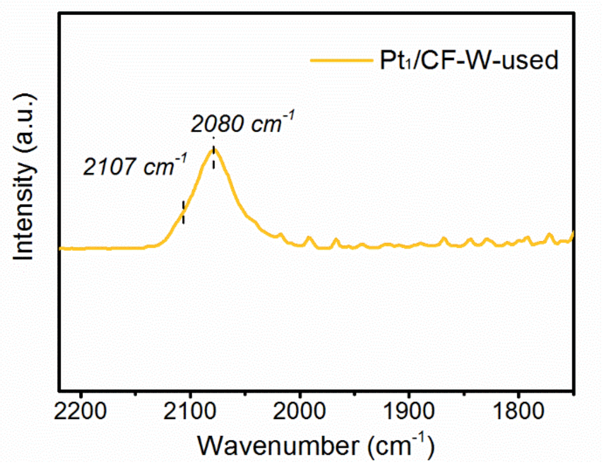

Figure S12. CO-DRFIT spectra of Pt_1/CF-W-used.

.

Figure S13

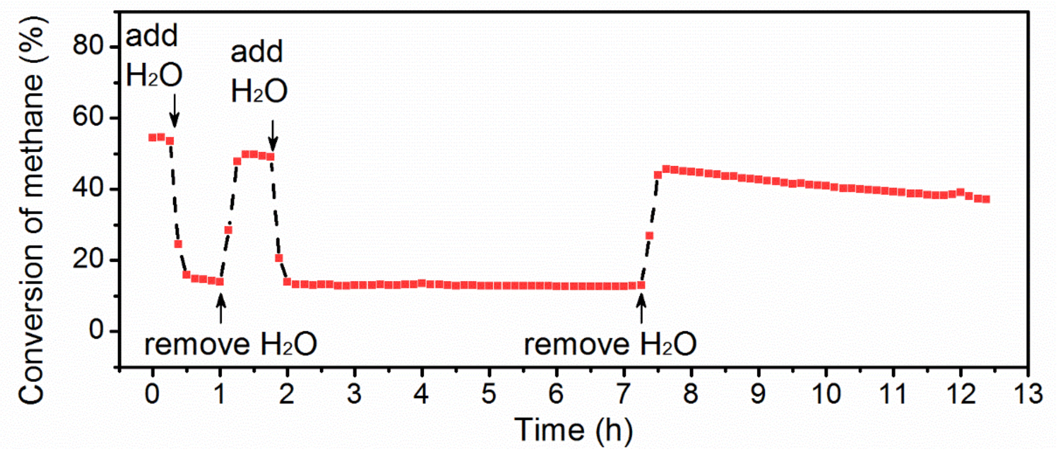

Figure S13. Methane conversion as a function of time on Pt_1/CF-W catalyst.

The reaction conditions: 0.5 vol% CH_4, 20 vol% O_2, 2.3 vol% H_2O (if exist) and 79.5 vol% N_2, GHSV = 10,000 ml/(g_{cat}·h), 300 °C.

Figure S14

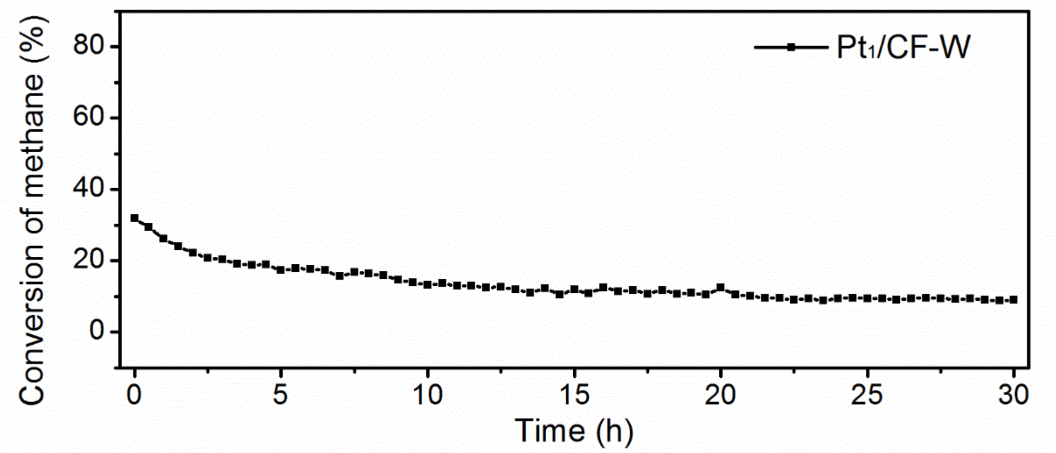

Figure S14. Methane conversion as a function of time on Pt_1/CF-W catalyst.

The reaction conditions: 0.5 vol% CH_4, 20 vol% O_2, and 79.5 vol% N_2, GHSV = 20,000 ml/(g_{cat}·h), 300 ºC.

Figure S15

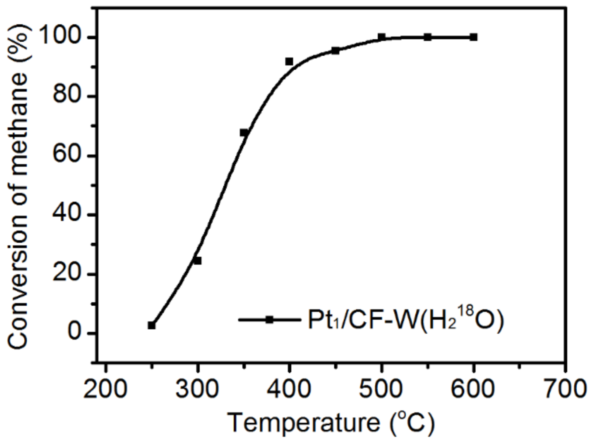

Figure S15. Light-off curves of CH_4 combustion over Pt_1/CF-W catalyst, which was pretreated by 1mL $H_2{}^{18}O$.

The reaction conditions: 0.5 vol% CH_4, 20 vol% O_2, and 79.5 vol% N_2, GHSV=20,000 ml/(g_{cat}·h).

Figure S16

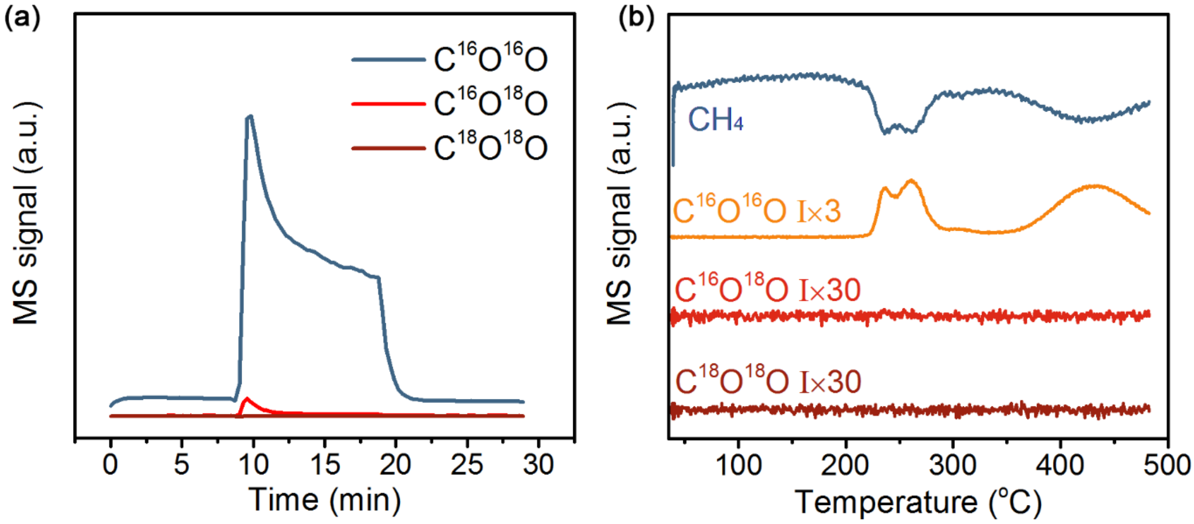

Figure S16. The O^{18} isotope labeling experiment: (a) The production of CO_2 on the Pt_1/CF-W($H_2{}^{18}O$) at 350 °C in the feed gas of 0.5 vol% CH_4, 20 vol% $^{16}O_2$ and 79.5 vol% N_2. (b) The production of CO_2 on the Pt_1/CF-W($H_2{}^{18}O$) in the CH_4-TPR.

Figure S17

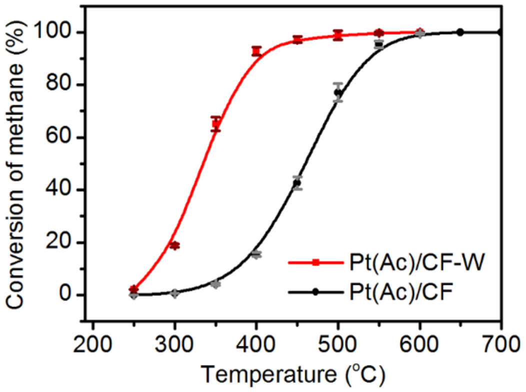

Figure S17. Light-off curves of CH_4 combustion over Pt(Ac)/CF and Pt(Ac)/CF-W catalysts using the $Pt(acac)_2$ precursor. The reaction conditions: 0.5 vol% CH_4, 20 vol% O_2, and 79.5 vol% N_2, GHSV = 20,000 ml/(g_{cat}·h). The error bars represent the standard deviation based on four repeated light-off experiment.

Figure S18

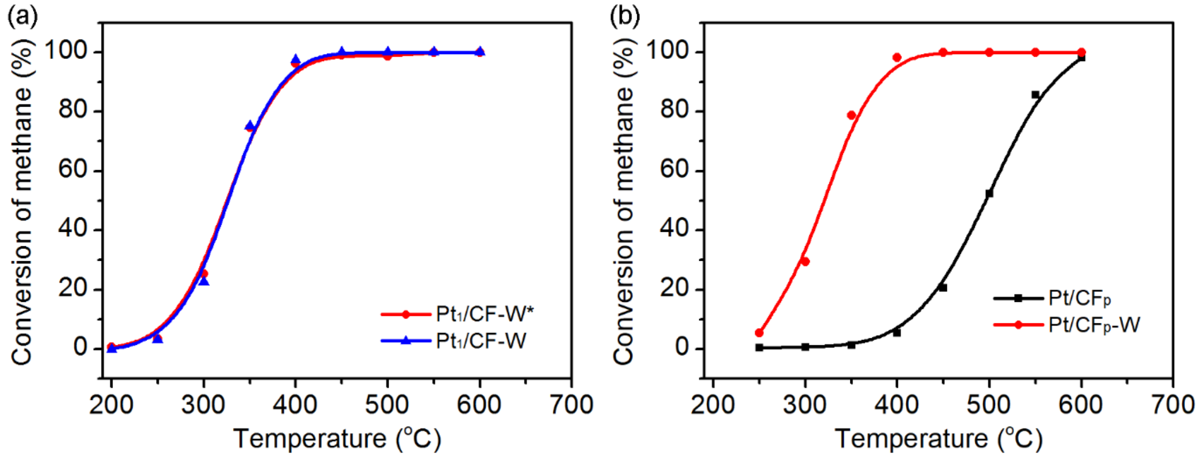

Figure S18. Light-off curves of CH_4 combustion over (a) Pt_1/CF-W* catalyst and (b) Pt_1/CF_p catalyst before and after water treatment.

The reaction conditions: 0.5 vol% CH_4, 20 vol% O_2, and 79.5 vol% N_2, GHSV=20,000 ml/(g_{cat}·h).

The Pt_1/CF-W* is the sample that pretreated by 1mL H_2O and directly dried without filtration.

The Pt_1/CF_p and Pt_1/CF_p-W are the samples prepared with purified $CoFe_2O_4$. In principle, 2 g $CoFe_2O_4$ was washed with 3 L water, dried at 80 °C overnight and then calcined at 500 °C for 4 h. The preparation of SAC and water treatment procedure were exactly the same as we did before.

Figure S19

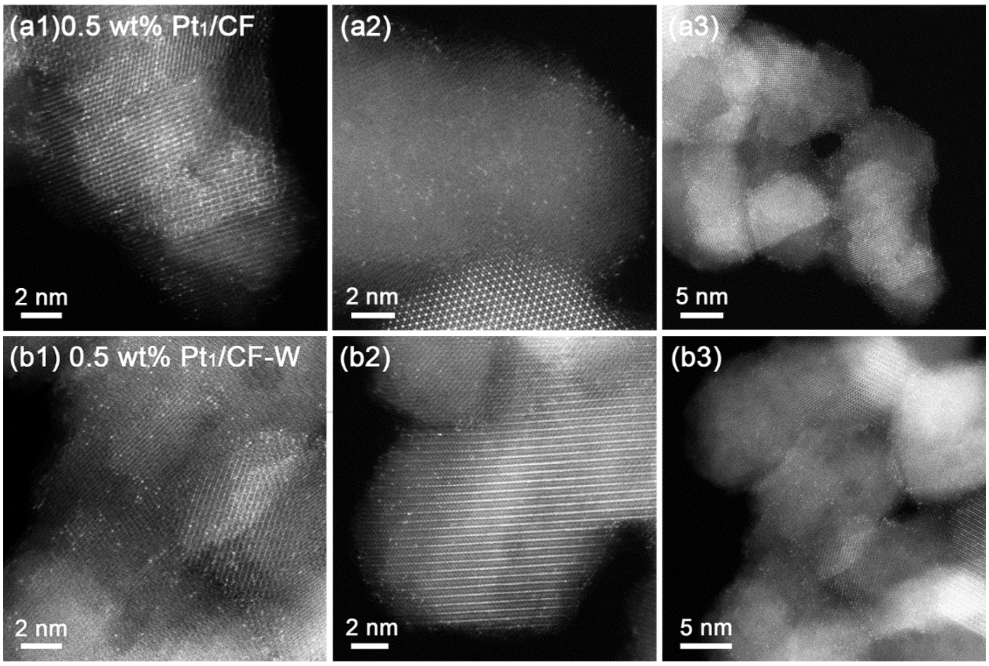

Figure S19. AC-HAADF-STEM images of 0.5 wt% Pt_1/CF catalysts before and after water treatment: (a) Pt_1/CF and (b) Pt_1/CF-W.

Figure S20

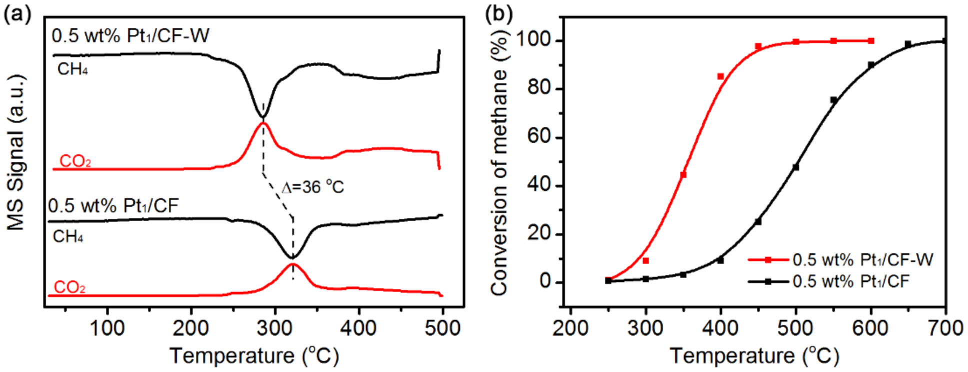

Figure S20. The performance of 0.5 wt% Pt_1/CF and Pt_1/CF-W catalysts: (a) CH_4-TPR under 1 vol% CH_4, 99 vol% He; (b) light-off curves of methane combustion. Reaction conditions: 0.5 vol% CH_4, 20 vol% O_2, N_2; GHSV = 20,000 ml/(g_{cat}·h)

Figure S21

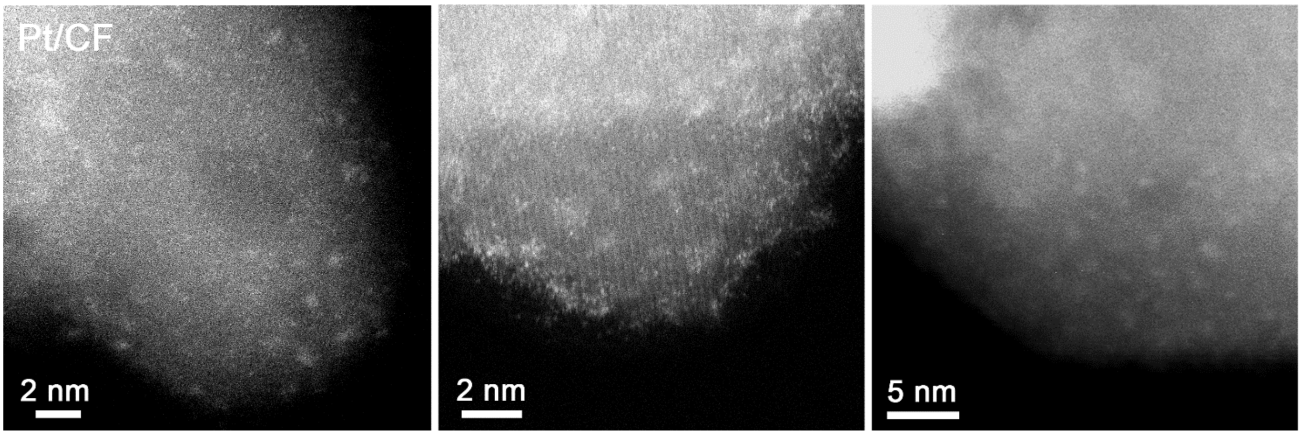

Figure S21. HAADF-STEM images of Pt/CF cluster catalyst.

The average size of Pt clusters on Pt/CF is 0.65 nm.

Figure S22

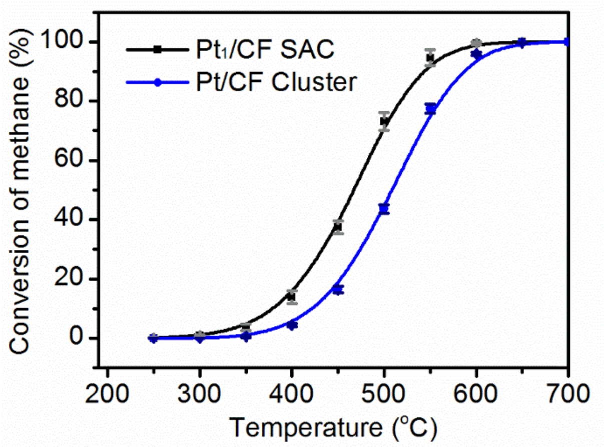

Figure S22. Light-off curves of CH_4 combustion over Pt_1/CF SAC and Pt/CF cluster catalysts. The reaction conditions: 0.5 vol% CH_4, 20 vol% O_2, and 79.5 vol% N_2, the total flow rate is 33.4 mL/min. The error bars represent the standard deviation based on four repeated light-off experiment.

Since the metal loading of Pt/CF cluster catalyst is 2.0 wt%, 50 mg Pt/CF catalysts were used in the activity test to keep the same Pt metal usage as Pt_1/CF. Obviously, the activity of Pt clusters are lower than the Pt single atoms, therefore the activity increase of Pt_1/CF SAC after water-soaking treatment cannot be ascribed to the formation of Pt small cluster, which is consistence with the observation on Pt/Fe_2O_3 catalyst in our previous report[1].

Figure S23

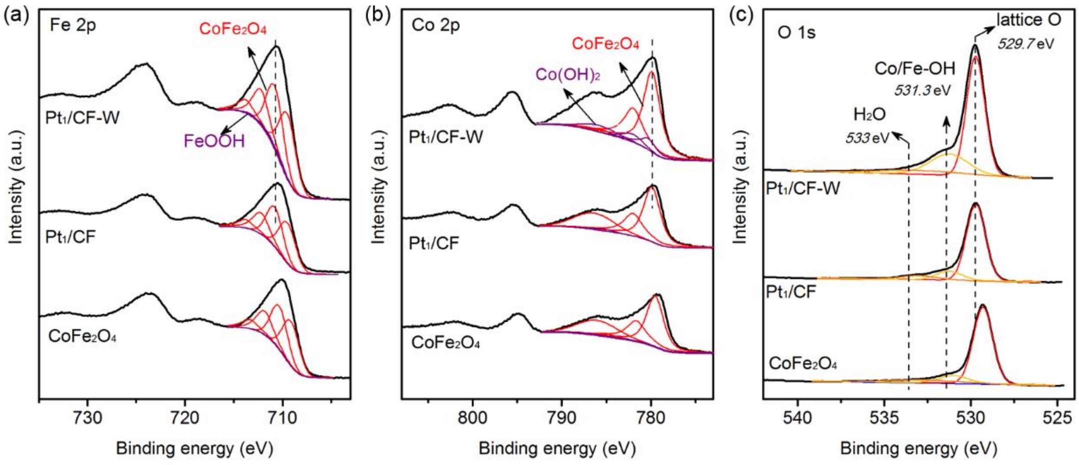

Figure S23. XPS spectra of $CoFe_2O_4$, Pt_1/CF and Pt_1/CF-W: (a) Fe 2p (b) Co 2p and (c) O 1s.

Figure S24

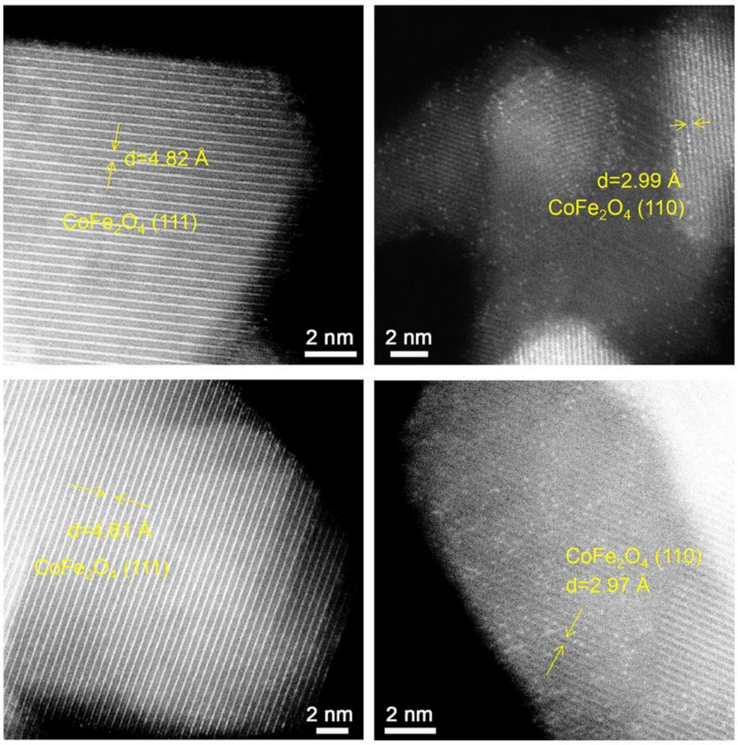

Figure S24. The distribution of Pt species on Pt_1/CF catalysts viewed on different facets.

Figure S25

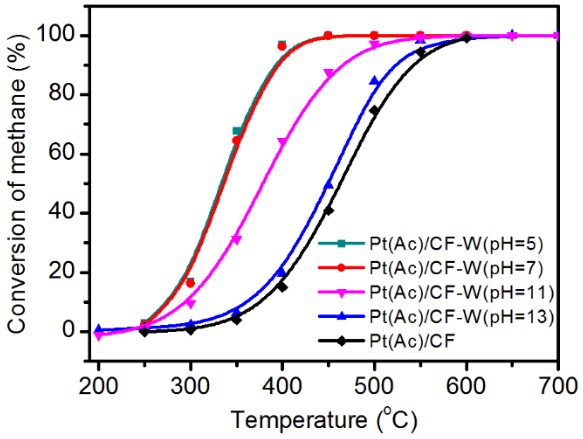

Figure S25. Light-off curves of CH_4 combustion over Pt(Ac)/CF catalysts pretreated with different aqueous solution. The reaction conditions: 0.5 vol% CH_4, 20 vol% O_2, and 79.5 vol% N_2; The total flow rate is 30 mL/min; GHSV = 20,000 ml/(g_{cat}·h).

The Pt_1/CF catalysts were pretreated with dilute NaOH or HNO_3 aqueous solution, respectively, and filtered and dried at 80 °C for 12 h. The activity of pretreated Pt_1/CF-W increased along with the concentration of H^+ of water increasing from 10^{-13} to 10^{-7} mol/L, but its activity cannot continuously increase with further increasing of H^+ concentration since H^+ must be coordinated with Pt-O-Fe to generate activated structure.

Figure S26

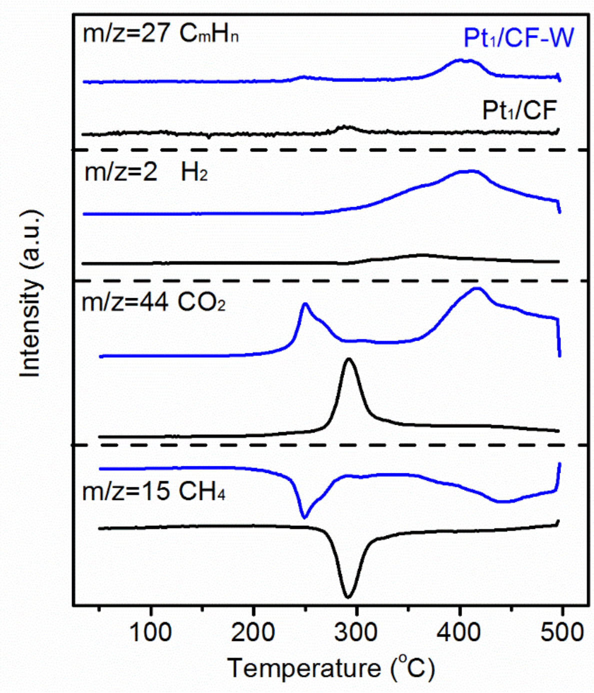

Figure S26. MS signal on the Pt_1/CF and Pt_1/CF-W in the CH_4-TPR experiment.

Figure S27

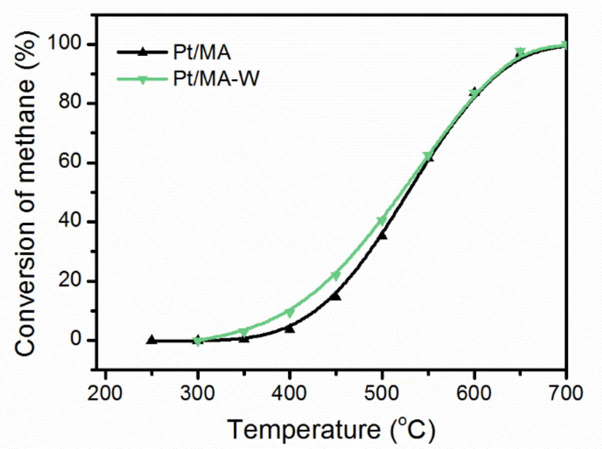

Figure S27. Light-off curves of CH_4 combustion over Pt/MA with and without water treatment catalysts. The reaction conditions: 0.5 vol% CH_4, 20 vol% O_2, and 79.5 vol% N_2; The total flow rate is 30 mL/min; GHSV = 40,000 ml/(g_{cat}·h).

Supplementary Tables

Table S1

Table S1. The quantity of CO adsorption on the Pt_1/CF and Pt_1/CF-W.

Sample	Metal loading (wt %)	CO adsorption quantity (μmol/g)	
		Theoretical	Actual
Pt_1/CF	1.0	51.3	2.282
Pt_1/CF-W	1.0	51.3	19.37

Table S2

Table S2. The quantity of H_2 consumption on the Pt_1/CF and Pt_1/CF-W.

Sample	Metal loading (wt %)	H_2 adsorption quantity (μmol/g)	
		Theoretical	Actual
Pt_1/CF	1.0	102	438
Pt_1/CF-W	1.0	102	537

Table S3

Table S3. The performances of Pt-based catalysts for CH_4 lean-burn combustion compared with literatures.

Entry	Sample	Pt wt%	Feed gas composition	GHSV ml/(g·h)	T_{50} °C	Reaction rate* (mmol mol_{metal}^{-1} s^{-1})	Ref.
1	Pt_1/CF	1.0	0.5% CH_4, 20% O_2, N_2	20,000	510	0.3 (300 °C)	this work
2	Pt_1/CF-W	1.0	0.5% CH_4, 20% O_2, N_2	20,000	325	-	this work
				80,000	-	16.3 (300 °C)	this work
3	Pt_1/CF-W	0.5	0.5% CH_4, 20% O_2, N_2	40,000	-	14.8 (300 °C)	this work
4	0.5H_2O_2-Pt_1/R-Mn_2O_3-5D	0.82	1% CH_4, 18% O_2, He	20,000	518	4.7 (400 °C)	[2]
5	Pt/Al_2O_3	1.8	0.2% CH_4, 10% O_2, N_2	400,000	625	4.0 (525 °C)	[3]
6	Pt/Al_2O_3	0.92	0.5% CH_4, 2% O_2, N_2	-	Above 500	-	[4]
7	Pt@CeO_2 (13.8 nm)	1.0	1% CH_4, 2% O_2, N_2	555,555	560	134 (450 °C)	[5]
8	CNR@Pt@CNP	2.18	1% CH_4, 20% O_2, N_2	240,000	550	26.6 (450 °C)	[6]
9	SO_2 treated Pt/CeO_2	4	0.05% CH_4, 8% O_2, Ar	15,000 h^{-1}	358	0.9 (300 °C)	[7]
10	Pt/Alumina	1.5	0.3% CH_4, 16% O_2, He	78,000	>350	3.0 (300 °C)	[8]
11	Pt/δ-Al_2O_3 (N0)	1.5	1% CH_4, 4% O_2, He	-	410	2.38 (300 °C)	[9]
12	Pt/ZrO_2	1.0	0.2% CH_4, 10% O_2, He	30,000	369	0.63 (300 °C)	[10]
13	Pt_1/CeO_2-Al_2O_3-300 reduction	1.0	1% CH_4, 4% O_2, He	200,000	297	67.7 (250 °C)	[11]
14	Pd/θ-Al_2O_3	1.0	0.5% CH_4, 2% O_2, N_2	200,000	295	100.0 (300 °C)	[12]
15	Pd@CeO_2/H-Al_2O_3	1.0	0.5% CH_4, 2% O_2, N_2	200,000	250	23.4 (250 °C)	[13]
16	Pd/Na-MOR	0.99	1% CH_4, 4% O_2, N_2	70,000	335	4.33 (275 °C)	[14]
17	Al_2O_3-Pd/$MgAl_2O_4$-A72	0.93	0.5% CH_4, 10% O_2, N_2	40,000	337	10.4 (300 °C)	[15]
18	Pd-Ce NW @SiO_2	1.5	1% CH_4, 21% O_2, N_2	36,000	290	9.65 (275 °C)	[16]
19	1Pd/2Pt@CeO_2	1.0 (Pd)	1% CH_4, 10% O_2, N_2	298,800	-	51.0 (300 °C) based on Pd	[17]
20	$LaFeO_3$-Pd/$MgAl_2O_4$	0.53	0.5% CH_4, 5% O_2, He	72,000	-	3.7 (300 °C)	[18]

* The reaction rate was calculated using the activity data obtained with CH_4 conversion below 15%

Table S4

Table S4. The best-fitted EXAFS results of Pt_1/CF catalysts.

	Shell	CN	R (Å)	σ^2 (10^{-2} Å^2)	ΔE_0 (eV)	r-factor (%)
Pt foil	Pt-Pt	12	2.76	0.004	3.2	0.004
PtO_2	Pt-O	6.0	2.00	0.002	3.5	0.005
	Pt-Pt	12	3.11	0.007	3.5	0.005
Pt_1/CF	Pt-O	4.8	1.99	0.003	5.1	0.016
	Pt-Fe	4.1	3.02	0.01	5.1	0.016
Pt_1/CF-W	Pt-O	4.4	2.00	0.002	6.7	0.009
	Pt-Fe	4.8	3.05	0.011	6.7	0.009

CN is the coordination number for the absorber–backscatterer pair, R is the average absorber-backscatterer distance, σ^2 is the Debye-Waller factor, and ΔE_0 the inner potential correction. The accuracies of the above parameters are estimated as CN, ±20%; R, ±1%; σ^2, ± 20%; ΔE_0, ±20%. The data range used for data fitting in k-space (Δk) and R-space (ΔR) are 3.0–12.0 Å^{-1} and 1.0–3.1 Å, respectively.

Table S5

Table S5. XPS analysis of the Pt, O, Fe and Co properties on the surface of samples.

Sample	O (%)			Fe (%)		Co (%)	
	H_2O	Fe/Co-OH	Lattice O	$CoFe_2O_4$	FeOOH	$CoFe_2O_4$	$Co(OH)_2$
$CoFe_2O_4$	-	0.12	0.88	~1	~99	~100	0
Pt/CF	-	0.13	0.87	~100	0	~99	~1
Pt/CF-W	-	0.28	0.72	~95	~5	~86	~14

Table S6

Table S6. The quantification of CH_4 and CO_2 in the CH_4-pulse experiment.

Pulse	The consumption of CH_4 (μmol/g_{cat})		The production of CO_2 (μmol/g_{cat})	
	Pt_1/CF	Pt_1/CF-W	Pt_1/CF	Pt_1/CF-W
1	3.49	39.18	0.16	12.35
2	1.85	32.39	0.24	12.83
3	2.76	32.36	0.24	12.07
4	2.17	35.23	0.26	11.32
5	1.80	35.48	0.20	10.31
6	1.42	33.45	0.17	9.50

Supplementary information for DFT calculation

1. *Pt/CF model*

According to the **Figure S24**, $CoFe_2O_4$ (110) is the surface where Pt single atoms anchor the most, therefore $CoFe_2O_4$ (110) is used for support in our simulations. Considering the symmetry of $CoFe_2O_4$ (110), there are only two kinds of sites that are possible for Pt to anchor. On the other hand, note that $CoFe_2O_4$ is ferrimagnetic at temperatures approximately below 650 K (Curie point), while in experiment calcination proceeds at 500 °C, Pt atom adsorption sites are thus tested without manually wavefunction symmetry breaking. Therefore, results shown in **Figure S28b** represent preference of Pt adsorption at temperatures above Curie point.

Because calcination proceeds under oxidative atmosphere, it is unreasonable to still have Pt-Fe bond in our model. One O atom between Pt and Fe is inserted to represent the result of oxidation, based on consideration that Fe occupies octahedral vacancy, can have O $CN = 6$ at maximum (**Figure S28d**). This modification makes Pt valence state and coordination environment more similar with that measured by XPS (**Figure 3b**) and EXAFS (**Figure 3d**), also makes the present site stabilize isolated Pt atom more than the other site.

To explore properties of catalyst at temperatures below Curie point, symmetry breaking is manually set in wavefunction initial guess procedure, in which Co and Fe atoms are set to have reversed spin orientation, shown in **Figure S28c**.

All structures in this work are visualized using the VESTA3[19] and Visual molecular dynamics (VMD).[20]

2. *Dissociated water adsorbed and water molecule adsorbed models tests*

5 structures were built respectively for dissociated water adsorbed and water molecule adsorbed Pt/CF(110) model. Optimized adsorbed structures are shown in **Figure S29** and **S30**. Suffix "d" indicates water dissociative adsorption mode and "m" indicates water molecule adsorption mode.

3. *Bader charge and Mayer bond order analysis*

Bader charge analysis is based on Atom-In-Molecule (AIM) picture, which is performed using Bader Charge Analysis V1.04 software[21-24]. Mayer bond order analysis is performed using Multiwfn-3.8-dev software[25], the grid spacing value is set to 0.4 Bohr after convergence test (**Figure S31**).

Figure S28

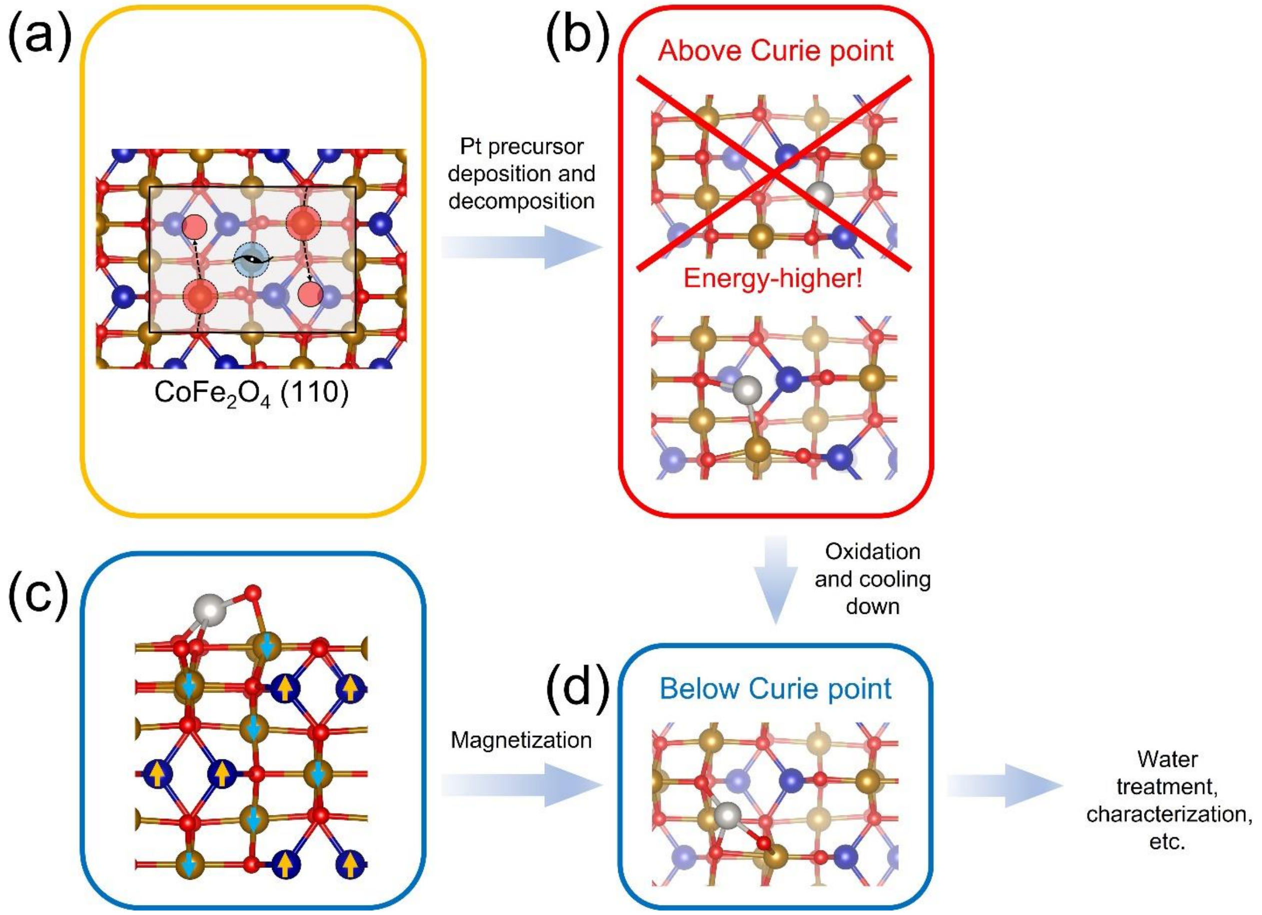

Figure S28. (a) Symmetry of $CoFe_2O_4$ (110) surface, C_2 rotation axis is marked at the center of semi-transparent rectangle, which highlights a centrosymmetric region. (b) Adsorption sites evaluation (calcination above Curie point). The upper is structure optimized from the one where Pt is placed at the position of blue circle in (a), the lower is optimized from the one where Pt is placed elsewhere (including red circles). The upper has energy higher than that of the lower for 14.71 eV. In these optimization tasks, support is considered to have zero magnetization. (c) Ferrimagnetic spin-alignment set in wavefunction initial guess for optimizing structures studied at temperatures below Curie point. (d) Structure obtained after O-insertion and optimization with support in ferrimagnetic phase, denoted as Pt/CF(110). Colors of atoms: O: red, Fe: brown, Co: blue, Pt: silver.

Figure S29

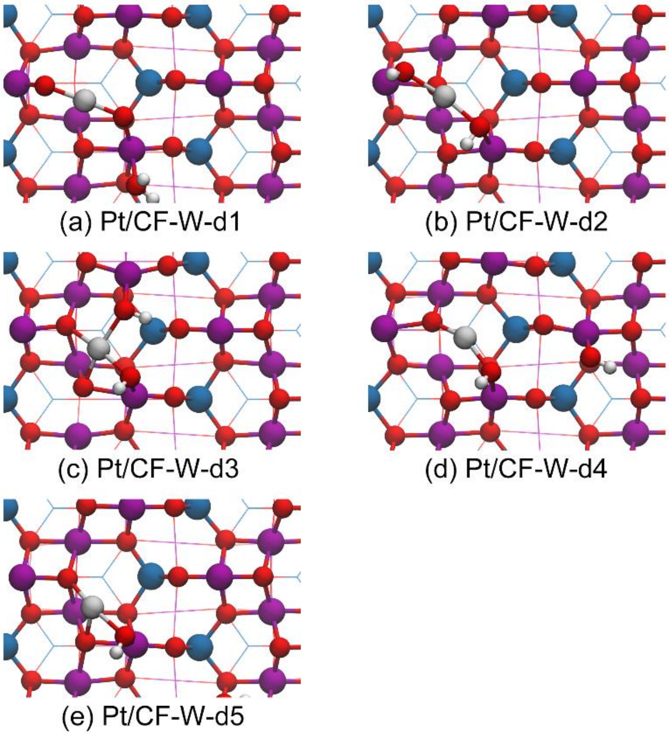

Figure S29. Structures of Pt/CF-W-d models: (a) Pt/CF-W-d1, (b) Pt/CF-W-d2, (c) Pt/CF-W-d3, (d) Pt/CF-W-d4 and (e) Pt/CF-W-d5.

“d” indicates water dissociative adsorption. Colors of atoms: H: white, O: red, Fe: purple, Co: blue, Pt: silver.

Figure S30

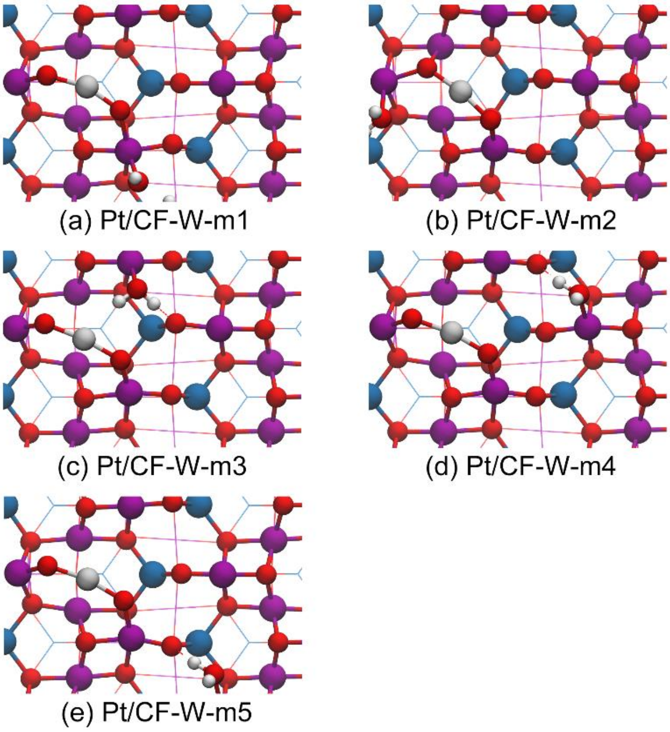

Figure S30. Structures of Pt/CF-W-m models: (a) Pt/CF-W-m1, (b) Pt/CF-W-m2, (c) Pt/CF-W-m3, (d) Pt/CF-W-m4 and (e) Pt/CF-W-m5.

“m” indicates water molecule adsorption. Colors of atoms: H: white, O: red, Fe: purple, Co: blue, Pt: silver.

Figure S31

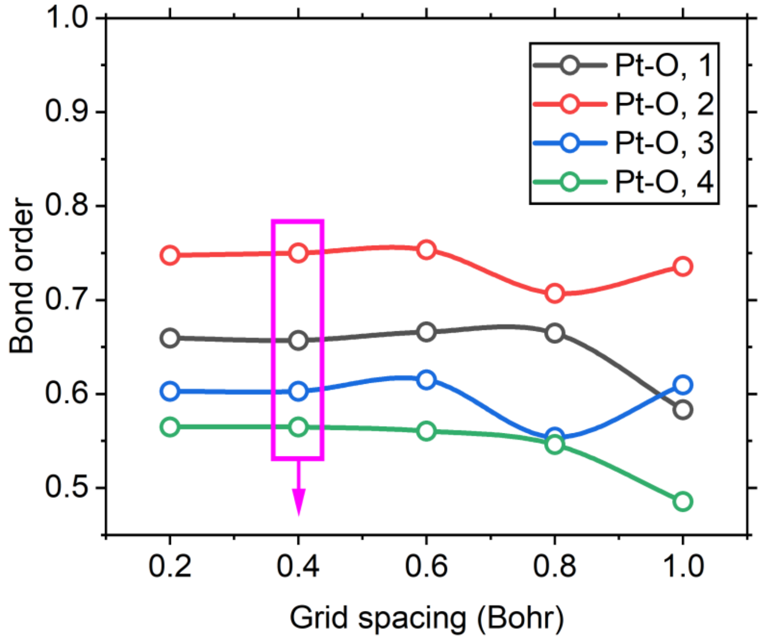

Figure S31. Gird spacing convergence test performed on Pt/CF(110)-W structure. As shown, 0.4 is a good choice for obtaining converged results balanced with computation cost.

Table S7

Table S7. Summary of simulation results of all models built in this work

Model	Energy/a.u.	Bader charge I*/e	Bader charge II*/e
Pt/CF	-	0.87	0.91
Pt/CF-W-d1	-11108.44	0.65	0.73
Pt/CF-W-d2	-11107.77	0.63	0.66
**Pt/CF-W-d3**	**-11108.63**	**0.84**	**0.89**
Pt/CF-W-d4	-11107.86	0.43	0.48
Pt/CF-W-d5	-11108.37	0.66	0.71
Pt/CF-W-m1	-11107.82	0.61	0.71
Pt/CF-W-m2	-11107.70	0.57	0.64
Pt/CF-W-m3	-11107.71	0.52	0.59
Pt/CF-W-m4	-11107.99	0.54	0.61
Pt/CF-W-m5	-11107.91	0.56	0.62

*Two kinds of charge distribution were used to calculate Bader charge[21-24]. For Bader I, only electron density is partitioned, for Bader II, total charge density is partitioned. Although charges obtained from these two methods are not totally the same, they still show identical tendencies across structures.

Supplementary References

1. Lang, R. et al. Non defect-stabilized thermally stable single-atom catalyst. *Nat. Commun.* **10**, 234-243 (2019).
2. Yan, D., Chen, J. & Jia, H. Temperature-Induced structure reconstruction to prepare a thermally stable single-atom platinum catalyst. *Angew. Chem. Int. Ed.* **59**, 13562-13567 (2020).
3. Wilburn, M.S. & Epling, W.S. Sulfur deactivation and regeneration of mono- and bimetallic Pd-Pt methane oxidation catalysts. *Appl. Catal. B: Environ.* **206**, 589-598 (2017).
4. Goodman, E.D. et al. Uniform Pt/Pd bimetallic nanocrystals demonstrate platinum effect on palladium methane combustion activity and stability. *ACS Catal.* **7**, 4372-4380 (2017).
5. Lee, S., Seo, J. & Jung, W. Sintering-resistant Pt@CeO_2 nanoparticles for high-temperature oxidation catalysis. *Nanoscale* **8**, 10219-10228 (2016).
6. Zhang, Z. et al. Thermally stable sandwich-type catalysts of Pt nanoparticles encapsulated in CeO_2 nanorod/CeO_2 nanoparticle core/shell supports for methane oxidation at high temperatures. *RSC Adv.* **6**, 40323-40329 (2016).
7. Kylhammar, L., Carlsson, P.-A. & Skoglundh, M. Sulfur promoted low-temperature oxidation of methane over ceria supported platinum catalysts. *J. Catal.* **284**, 50-59 (2011).
8. Gracia, F.J., Miller, J.T., Kropf, A.J. & Wolf, E.E. Kinetics, FTIR, and controlled atmosphere EXAFS study of the effect of chlorine on Pt-supported catalysts during oxidation reactions. *J. Catal.* **209**, 341-354 (2002).
9. Marceau, E., Lauron-Pernot, H. & Che, M. Influence of the metallic precursor and of the catalytic reaction on the activity and evolution of Pt(Cl)/δ-Al_2O_3 catalysts in the total oxidation of methane. *J. Catal.* **197**, 394-405 (2001).
10. Torralba, R. et al. Total oxidation of methane over sulfur poisoning resistant Pt/ZrO_2 catalyst: Effect of Pt^{2+}-Pt^{4+} and Pt^{2+}-Zr^{4+} dipoles at metal-support interface. *Catal. Lett.* **151**, 1592-1603 (2021).
11. Jeong, H. et al. Controlling the oxidation state of Pt single atoms for maximizing catalytic activity. *Angew. Chem. Int. Ed.* **59**, 20691-20696 (2020).
12. Murata, K. et al. The metal-support interaction concerning the particle size effect of Pd/Al_2O_3 on methane combustion. *Angew. Chem. Int. Ed.* **56**, 15993-15997 (2017).
13. M., C. et al. Exceptional activity for methane combustion over modular Pd@CeO_2 subunits on functionalized Al_2O_3. *Science* **337**, 713-717 (2012).
14. Petrov, A.W. et al. Stable complete methane oxidation over palladium based zeolite catalysts. *Nat. Commun.* **9**, 2545 (2018).
15. Yang, J. et al. Hydrothermally stable irreducible oxide-modified Pd/$MgAl_2O_4$ catalyst for methane combustion. *Angew. Chem. Int. Ed.* **59**, 18522-18526 (2020).
16. Peng, H. et al. Confined ultrathin Pd-Ce nanowires with outstanding moisture and SO_2 tolerance in methane combustion. *Angew. Chem. Int. Ed.* **57**, 8953-8957 (2018).
17. Xiong, H. et al. Engineering catalyst supports to stabilize PdO_x two-dimensional rafts for water-tolerant methane oxidation. *Nat. Catal.* **4**, 830-839 (2021).
18. Onn, T.M. et al. Smart Pd catalyst with improved thermal stability supported on high-surface-area $LaFeO_3$ prepared by atomic layer deposition. *J. Am. Chem. Soc.* **140**, 4841-4848 (2018).
19. Momma, K. & Izumi, F. VESTA 3 for three-dimensional visualization of crystal, volumetric and morphology data. *J. Appl. Crystallogr.* **44**, 1272-1276 (2011).

20. W., H., Dalke, A. & Schulten, K. VMD-Visual Molecular Dynamics. *J. Molec. Graphics* **14**, 33-38 (1996).

21. Tang, W., Sanville, E. & Henkelman, G. A grid-based Bader analysis algorithm without lattice bias. *J. Phys.: Condens. Matter* **21**, 084204 (2009).

22. Sanville, E., Kenny, S.D., Smith, R. & Henkelman, G. Improved grid-based algorithm for Bader charge allocation. *J. Comput. Chem.* **28**, 899-908 (2007).

23. Henkelman, G., Arnaldsson, A. & Jónsson, H. A fast and robust algorithm for Bader decomposition of charge density. *Comp. Mater. Sci.* **36**, 354-360 (2006).

24. Yu, M. & Trinkle, D.R. Accurate and efficient algorithm for Bader charge integration. *J. Chem. Phys.* **134**, 064111 (2011).

25. Lu, T. & Chen, F. Multiwfn: A Multifunctional Wavefunction Analyzer. *J. Comput. Chem.* **33**, 580-592 (2012).
